# Insulin versus Lipid Emulsion in a Rabbit Model of Severe Propranolol Toxicity: A Pilot Study

**DOI:** 10.1155/2011/361737

**Published:** 2011-03-31

**Authors:** Martyn Harvey, Grant Cave, Daniel Lahner, Jan Desmet, Gaynor Prince, Gary Hopgood

**Affiliations:** ^1^Department of Emergency Medicine, Waikato Hospital, Pembroke Street, Hamilton 3204, New Zealand; ^2^Emergency Department, Hutt Hospital, High Street, Lower Hutt 5011, New Zealand; ^3^Department of Anesthesia, Waikato Hospital, Pembroke Street, Hamilton 3204, New Zealand

## Abstract

*Background and objective*. Beta-blocker overdose may result in intractable cardiovascular collapse despite conventional antidotal treatments. High dose insulin/glucose (ING), and more recently intravenous lipid emulsion (ILE), have been proposed as potentially beneficial therapies in beta blocker intoxication. We compare efficacy of the novel antidotes ING, with ILE, in a rabbit model of combined enteric/intravenous propranolol toxicity. *Methods*. Sedated, mechanically ventilated and invasively monitored New Zealand White rabbits underwent mini-laparotomy and enterostomy formation with 40 mg/kg propranolol instilled into the proximal small bowel. At 30 minutes propranolol infusion was commenced at 4 mg/kg/hr and continued to a target mean arterial pressure (MAP) of 50% baseline MAP. Animals were resuscitated with insulin at 3 U/kg plus 0.5 g/kg glucose (ING group), or 10 mL/kg 20% Intralipid (ILE group). *Results*. Rate pressure product (RPP; RPP = heart rate × mean arterial pressure) was greatest in the ING group at 60 minutes (*P* < .05). A trend toward greater heart rate was observed in the ING group (*P* = .06). No difference was observed in survival between groups (4/5 ING versus 2/5 ILE; *P* = .524). *Conclusions*. High dose insulin resulted in greater rate pressure product compared with lipid emulsion in this rabbit model of severe enteric/intravenous propranolol toxicity.

## 1. Introduction

Beta-adrenergic-blocking drugs are invaluable in treating a range of cardiovascular and noncardiovascular medical conditions. Accidental and intentional overdose may, however, result in intractable cardiovascular collapse. Reversal of bradycardia, negative inotropism, and restoration of critical organ perfusion are immediate goals of treatment. Propranolol is a lipophilic, nonselective, beta-blocker with additional sodium channel blocking activity [1]. These unique features are thought to contribute to the increased mortality associated with propranolol intoxication when compared with poisoning from other beta-blocking agents [2].

Numerous therapies have been advocated as useful in severe beta-blocker poisoning including volume expansion, atropine, calcium, cardiac pacing, infusion of inotropic and vasoactive medications, gastrointestinal decontamination, and charcoal hemodiafiltration [3]. Glucagon is furthermore known to exert a positive inotropic and chronotropic effect on the myocardium by stimulating adenyl cyclase through a separate receptor to that of catecholamines [4, 5]. Although widely employed, however, glucagon has failed when administered as sole antidotal therapy in a number of clinical reports of beta-blocker intoxication [6–8].

More recently a number of novel therapies have been forwarded as potentially useful in beta-blocker overdose. Levosimendan, a calcium sensitizer, has been demonstrated to improve survival when compared with both placebo and dopamine in a swine model of severe propranolol toxicity [9]. High-dose insulin/dextrose euglycaemia has additionally been demonstrated superior to both glucagon and adrenaline, and combination vasopressin and adrenaline, in canine [10] and porcine [11] models of propranolol intoxication, respectively. Insulin use has been employed in greater than 68 reported cases of calcium channel blocker/beta-blocker overdose with largely positive cardiodynamic, and hemodynamic responses [3], albeit few of these reported ING applications in single agent beta-blocker poisoning.

Our study group has additionally demonstrated improvements in hemodynamic performance with infusion of lipid emulsions in propranolol-induced hypotension in whole rabbits [12]. No benefit was demonstrated in similar models of toxicity from the more hydrophilic beta-blockers atenolol [13], and metoprolol [14]. Sequestration of highly lipophilic toxins to an expanded intravascular lipid phase and augmentation of free fatty acid metabolism have been forwarded as potential beneficial mechanisms of action of intravenous lipid emulsions [15–17]. Demonstrated efficacy for ILE in cardioactive agents of elevated lipophilicity appears consistent with the former thesis.

A paucity of comparative data exists to guide the clinician faced with a patient in profound cardiovascular collapse secondary to propranolol poisoning, when choosing to institute one or more of these novel therapies. Accordingly this study was undertaken to evaluate the effects of insulin and glucose (ING) and intravenous lipid emulsion (ILE) in an animal model of propranolol toxicity. Specifically we determined to compare hemodynamic performance following ING versus ILE therapy in an intact rabbit model of propranolol-induced shock.

## 2. Methods

This study was performed at the Ruakura Animal Research Centre, Hamilton, New Zealand. All study protocols were reviewed and approved by the Ruakura Animal Ethics Committee. Animal utilization was in accord with institutional guidelines for ethical animal experimentation.

### 2.1. Experimental Model

Fourteen adult New Zealand White Rabbits (age 95–114 days) of mixed gender were studied. Prior to study animals were housed in single-sex enclosures with no chance of pregnancy. Unfettered access to feed and water was permitted until the day utilization. 

On the day of study rabbits were sedated with ketamine (Mayne Pharma Ltd, New Zealand) at 50 mg/kg, and xylazine (Bayer HealthCare, Germany) at 4 mg/kg via intramuscular injection. Animals were then placed on a surgical board and underwent venous cannulation of the marginal vein of the ear. Intravenous Ketamine bolus (10 mg) was administered prior to subsequent invasive procedures. Three lead ECGs were sited, with continuous monitoring of standard limb lead two. 

Formal tracheostomy was preformed with 3.0 mm internal diameter endotracheal tube secured by taping, and mechanical ventilation initiated with 100% oxygen titrated to 10 cm H_2_O inspiratory pressure at 27 breaths per minute (Nuffield series 200 paediatric ventilator, Penlon Ltd, Abington, England). Inspiratory : expiratory ratio was set at 0.2 : 2.0. End tidal CO_2_ was assessed with continuous colour-change capnography (C-CO_2_, Vital Signs Colorado Ltd, USA) with CO_2_ maintained in the 2–5% range prior to induction of toxicity. Vecuronium (Pharmaco Ltd, New Zealand) at 0.1 mg/kg was administered following establishment of mechanical ventilation, and repeated at 20 minute intervals to maintain paralysis. 

Blunt dissection was employed to expose the common carotid artery on the right before cannulation with a 20 G saline filled vascular catheter advanced to the proximal aorta. This was connected in standard fashion (Edwards Lifesciences pressure transducer, Irvine, CA, USA) to Hewlett-Packard 78834A neonatal monitor for continuous assessment of invasive arterial blood pressure. Minilaparotomy was preformed with identification of the junction of the first and second parts of the duodenum. Enterostomy (5 mm) was created and a catheter tipped feeding tube passed distally for a distance of 25 cm into the small bowel. A ten-minute period of stabilization was afforded following completion of invasive procedures at which time baseline hemodynamic metrics were obtained.

### 2.2. Induction Toxicity

Propranolol (*Cardinol*, Pacific Pharmaceuticals, Auckland, New Zealand) tablets at 40 mg/kg were crushed and added to 20 mL/kg sterile water. This was instilled via the enteric feeding tube during gradual withdrawal, ensuring uniform delivery of slurry over the instrumented small bowel. The tube was then removed, and enterostomy and laparotomy closed. 

At 30 minutes propranolol 0.3 mg/kg (*Inderal*, Wyeth Pharmaceuticals Inc, Phil, USA) was injected intravenously and thereafter continued at 4 mg/kg/hour until mean arterial pressure (MAP) equaled 50% baseline MAP. At this point propranolol infusion was discontinued.

### 2.3. Resuscitation Protocol

Animals were entered into intravenous lipid emulsion (ILE) or insulin and glucose (ING) treatment arms (*n* = 5 all groups) according to prior randomization. At the defined point of toxicity animals in the ILE group received 10 mL/kg 20% Lipid emulsion (Intralipid, Fresenius Kabi AB, Sweden) injected over a five minute interval. Animals in the IN/G group received 3 u/kg insulin (*Actrapid, *Novo Nordisk AS, Denmark), 0.5 g/kg glucose, and 7 mL/kg 0.9% saline solution over five minutes. All rabbits were monitored for 60 minutes following administration of study agents with acquisition of hemodynamic metrics at one minute intervals.

In the event of cardiovascular collapse (defined by mean arterial pressure of less than or equal to 20 mm Hg) ILE-treated animals received a further 3 mL/kg 20% Intralipid infused over one minute. Adrenaline (Hospira, New Zealand) 100 mcg/kg was administered and repeated at 5 minute intervals, and external chest compressions (approximating 30% anterior-posterior chest wall diameter at 180 beats per minute) commenced. Mechanical ventilation continued unabated. External chest compressions were discontinued for five seconds every minute to assess native hemodynamic parameters. Return of spontaneous circulation (ROSC) was defined by an MAP of greater than or equal to 50 mm Hg with restoration of perfusing native heart rhythm, for 120 seconds or longer. Animals failing to exhibit ROSC at ten minutes following onset of cardiovascular collapse underwent crossover treatment to receive 3 u/kg insulin, 0.5 g/kg glucose, and 2 mL/kg 0.9% saline solution over one minute. Basic life support (BLS) resuscitative measures and bolus adrenaline administration were continued until termination of the monitoring interval. 

Animals from the ING group exhibiting cardiovascular collapse received a further 3 u/kg insulin, 0.5 g/kg glucose, and 2 mL/kg 0.9% saline solution over one minute. BLS resuscitation and bolus adrenaline were likewise continued for ten minutes. Animals failing to exhibit ROSC then underwent crossover to receive 3 mL/kg 20% Intralipid infused over one minute. In similar fashion BLS resuscitation and repeated bolus adrenaline was continued to termination of the monitoring interval.

At the termination of study protocols all surviving animals were killed with intravenous pentobarbitone (300 mg/kg) overdose. Necropsy was performed to confirm appropriate positioning of endotracheal tube and vascular catheters, and absence of peritoneal hemorrhage.

### 2.4. Data Acquisition

Heart rate and invasive blood pressure (systolic, diastolic, mean arterial pressure (MAP)) were recorded directly from the monitoring system to standardized data collection template at one-minute intervals. Heart rate (HR) was recorded to the nearest beat per minute and blood pressure to the nearest mmHg. Rate pressure product (RPP; RPP = HR × MAP) was adopted as the key measure of cardiovascular performance given incorporation of the depressant effect of propranolol on both cardiac chronotropic and inotropic states.

### 2.5. Statistical Analysis

Statistical analysis of all variables was performed using GraphPad Prism 5 (GraphPad Software, San Diego, CA). Following assessment for normality with the Kolmogorov-Smirnov test animal characteristics and baseline hemodynamic parameters were compared via Student's *t*-testing. All hemodynamic metrics were compared across time by two-way repeated measures analysis of variance (ANOVA) with Bonferroni posttesting when significance was achieved (*α* < 0.05). Survival rates were compared as a secondary variable of interest using Fisher's exact statistics. All metrics are presented as mean (SEM) unless otherwise stated. A two-tailed *P* < .05 was retained as statistically significant.

## 3. Results

Animal characteristics and baseline hemodynamic parameters are presented in Table 1. All metrics passed the Kolmogorov-Smirnov test for normality. There were no differences in any parameter between groups. 

**Figure 1:**
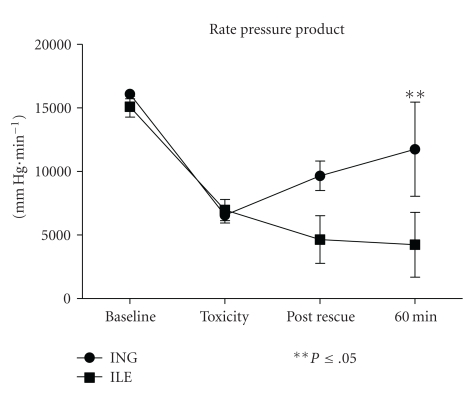
Rate pressure product (RPP) versus time.

Total propranolol dosing to toxicity was 41.9 (0.73) mg/kg in the ING group, and 41.9 (0.64) mg/kg in the ILE group. Intravenous propranolol was 1.92 (0.73) in the insulin group and 1.90 (0.64) in the ILE group. Rate pressure product served as the key measure of cardiac performance in this model and is displayed graphically in Figure 1. A trend toward greater RPP was observed in the insulin-treated group (*P* = .076; RMANOVA). A significant differential in RPP was observed at 60 minutes.

**Figure 2:**
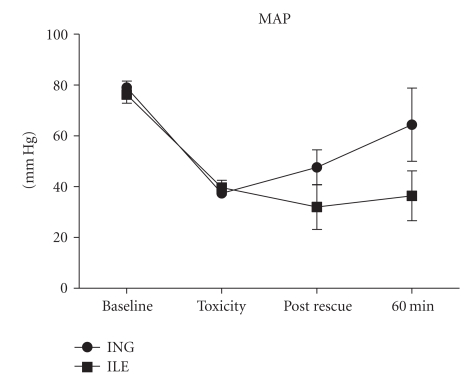
Mean arterial pressure (MAP) versus time.

**Figure 3:**
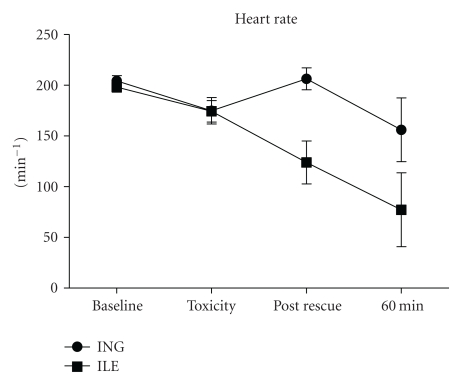
Heart rate versus time.

Mean arterial pressure and heart rate across time are presented graphically in Figures 2 and 3, respectively. No statistically significant difference was observed in MAP between groups (*P* = .106; RMANOVA). A trend toward greater heart rate in the insulin-treated group was observed (*P* = .06; RMANOVA). At toxicity all animals exhibited visible widening of ECG QRS duration consistent with the known sodium channel blocking effect of propranolol. Limitations in monitoring equipment however precluded capture and subsequent further interrogation of key ECG metrics. 

At termination of the study protocol one animal (1/5) from the insulin-treated group and three animals (3/5) from the ILE group were undergoing resuscitative treatment following manifest cardiovascular collapse (*P* = .524). No animal from either group exhibited ROSC following cardiovascular collapse with initial resuscitative therapy, or following crossover to the alternate treatment arm.

Necropsy revealed appropriate endotracheal tube, and vascular catheter positioning in all animals. No evidence of significant hemoperitoneum was observed in any animal.

## 4. Discussion

In this pilot model of severe propranolol intoxication, high-dose insulin (ING) therapy resulted in superior rate pressure product at 60 minutes when compared with intravenous lipid emulsion (ILE). Insulin administration was likewise associated with a trend toward greater amelioration of propranolol-induced bradycardia. No difference was observed in survival between groups, albeit the study was powered to detect all-or-none differences in categorical variables only. No animal from either grouping exhibited return of spontaneous circulation following cardiovascular collapse with ING or ILE resuscitative treatments administered individually or in combination.

In keeping with the goals of the present experiment (to evaluate ING and ILE in isolation) we did not employ either a placebo arm, or a standard treatment (atropine, calcium, glucagon, and volume expansion) arm, in the study protocol of this experiment. Similarly due to the preliminary nature of the study hypothesis and unique model utilized, no combination ING/ILE treatment arm was included as initial resuscitation. While these limitations mitigate against recommendation for clinical application, it would appear that ING is superior therapy to ILE in this model of severe propranolol poisoning.

Although incompletely elucidated, the beneficial effect of insulin in drug-induced shock may be due to its role in carbohydrate metabolism [10]. Under normal conditions myocytes prefer free fatty acids as substrate for generation of high-energy phosphate compounds. During drug-induced shock this preferred substrate is known to shift from free fatty acids to carbohydrates [17, 18]. Cardiac uptake and utilization of glucose is nevertheless inhibited during beta-blocker intoxication due to hypoinsulinaemia and acquired insulin resistance [19–21], effects reversed in the presence of insulin. Importantly improvements in cardiac function following insulin administration occur in the absence of increased myocardial work [18, 22]. In contrast treatment with calcium, glucagon, and catecholamine's all promote free fatty acid utilization, and subsequently increased myocardial work [18]. In addition to improving myocardial glucose utilization, insulin is furthermore known to increase levels of plasma calcium, improve the hyperglycaemic acidotic state associated with drug-induced shock, and exert an independent inotropic effect [23, 24]. This inotropic effect is heightened in stressed myocardium, and in beta-blocker cardiotoxicity in particular [25].

Clinical experience with insulin is favorable. High-dose insulin euglycaemia has been utilized in greater than 68 reports of calcium channel blocker and/or beta-blocker overdose with most authors reporting favorable cardiovascular performance with insulin administration [10]. While less frequent, the instances of insulin use in sole beta-blocker overdose similarly reflect improvement in reported hemodynamic metrics. 

Intravenous lipid emulsions have garnered recent interest as antidotal therapy in nonlocal-anaesthetic lipophilic drug toxidromes [26–28] following a decade of experimental and clinical use in high lipophilicity local anaesthetic cardiotoxicity [29, 30]. Two main hypotheses have been forwarded to explain the beneficial effects observed with ILE application [30]. In the first, lipid infusion serves to create an expanded plasma lipid phase (sink), into which highly lipophilic toxins are sequestered away from their pharmacologic sites of action. Expedited redistribution to less vital lipid rich tissues may additionally be associated with enhanced toxin carriage [31]. The second mechanism proposes augmented myocardial lipid metabolism with bolus free fatty acid administration overcoming pharmacologic impediments to fatty acid oxidation known to be associated with responsive toxic agents [30]. 

Limited experimental evidence with ILE in animal models of beta-blocker intoxication has suggested benefit with lipid application in agents of elevated lipophilicity. Bania and coworkers have demonstrated increased mean arterial pressure (MAP) in propranolol toxic rodents following ILE infusion [32]. Similarly our study group has demonstrated greater hemodynamic performance in rabbits resuscitated from propranolol-induced hypotension with ILE compared placebo [12], but nil benefit in similar models of the more hydrophilic beta-blockers atenolol [13] and metoprolol [14] thereby providing inferential support to the “lipid sink” hypothesis.

Clinical reports of ILE use in beta-blocker toxicity are emerging. In one case report ILE application resulted in dramatic improvements in hemodynamic performance with reversal of overt shock repeatedly in a patient suffering massive verapamil and atenolol overdose. The improvements were however nonsustained and the patient eventually died despite maximal conventional therapies, repeated ILE bolus, and intra-aortic balloon counterpulsation [33]. In a further report of atenolol self-poisoning a 46-year-old female received 100 mL of ILE over one hour resulting in stabilization of blood pressure following the development of symptomatic hypotension [34]. The patient survived. 

Two literature reports describe combined ING/ILE use in beta-blocker intoxication. A 48-year-old man was successfully resuscitated from nebivolol-induced bradyasystolic cardiac arrest with 100 mL 20% ILE following failure of conventional antidotal therapies. Subsequent transient hypertension was attributed to unopposed epinephrine effect on beta adrenergic receptors previously occupied by nebivolol, yet rendered unoccupied through proposed myocardial toxin washout in accord with lipid sink thesis. The patient subsequently received high-dose insulin cotherapy and survived [35]. In the second, a 31-year-old woman remained hypotensive following massive carvedilol ingestion despite volume expansion, high dose dopamine infusion, calcium chloride bolus's, glucagon, epinephrine infusion, and an intensive insulin regimen. Following ILE bolus and infusion blood pressure normalized and she was rapidly weaned from vasopressors and insulin [36]. Significantly carvedilol shares with propranolol the commonality of high lipid solubility (Log *P* carvedilol 3.29 [37]; propranolol 2.80 [38]).

In the context of the available experimental and clinical data, and on the basis of postulated beneficial mechanisms of action of the two agents, it would appear that high-dose insulin has demonstrated more consistent efficacy and clinical utility than ILE in beta-blocker intoxication. ILE has nevertheless been shown to affect recovery in clinical scenarios of cardiotoxicity attributable to beta-blocking agents of elevated lipophilicity, when conventional therapies—including high-dose insulin, have proven ineffectual. While clinical recommendation based on incomplete data is fraught, high-dose insulin therapy may be considered preferential to ILE in the majority of beta-blocker poisonings, with consideration given to ILE for intoxications characterized by greater lipophilicity of the offending agent.

Some features of the animal model employed in the present work warrant further mention. The decision to instrument the small bowel and deliver propranolol enterically represents a significant deviation from previous models which have employed continuous intravenous infusions of toxin as mimic for ongoing gastrointestinal absorption [11]. Enterostomy was nevertheless elected following initial dose ranging experiments with orogastric tube insertion (4 animals) that failed to result in overt pharmacologic effect within sufficient time frame to feasibly study. Given little is known about mesenteric blood flows and intestinal absorption of propranolol during intoxication-induced shock states, this was considered more likely to reflect the ongoing gastrointestinal absorption expected following oral overdose. Intravenous infusion was however retained to achieve a toxic hemodynamic endpoint in a timely fashion. 

In this study we elected intramuscular ketamine/xylazine combination as sedation in line with both institutional protocol, and previous demonstrated cardiovascular stability of these agents in similar models of toxicity [12–14]. Ketamine is nevertheless known to exhibit intrinsic sympathomimetic activity which may have partially mitigated the beta adrenergic blocking effects of propranolol. We are therefore unable to exclude a confounding effect of ketamine on observed outcomes. All animals however received a pharmacologically available preparation of propranolol, and behaved in a fashion consistent with beta-blocker intoxication.

This study is subject to a number of additional limitations. The study was by nature a preliminary pilot study aimed at establishing a model for future trials. The small number of animals in each group rendering the trial insufficiently powered to detect anything but larger differences in categorical variables (ROSC, survival). These data may however be gainfully employed to power future work with these metrics as primary outcome variables. The experiment was additionally performed by investigators unblinded to key study interventions. Acquisition of study metrics were however obtained directly from monitoring equipment to standardized data collection template in a systematic fashion.

The study furthermore failed to obtain blood gas analysis at baseline, toxicity, or 60 minutes. As such we are unable to compare important physiologic parameters prior to, and following, propranolol infusion. All animals however received identical care, and received mechanical ventilation with identical settings. Similarly we have been unable to collate serum glucose and potassium concentrations during the course of the experimental protocol. Significant hypoglycaemia is however unlikely given the glucose bolus administered concurrently with insulin. Finally we adopted 3 IU/kg as initial bolus in the ING group. This represents three times the dosage generally employed (recommendation 1 IU/kg [3]) yet afforded greatest potential for observing a positive effect on outcomes.

## 5. Conclusion

In this rabbit model of severe enteric/intravenous propranolol toxicity high-dose insulin resulted in greater hemodynamic recovery compared with intravenous lipid emulsion. Further experimental work is indicated to guide clinical recommendations for lipid emulsion therapy in beta-blocker poisoning.

## Figures and Tables

**Table 1:** Animal characteristics and baseline parameters according to group.

	ING (*n* = 5)	ILE (*n* = 5)
Age (days)	110 (4)	104 (5)
Gender (M : F)	4 : 1	3 : 2
Weight (g)	3200 (32)	3094 (94)
Baseline heart rate (min^−1^)	204 (5)	198 (5)
Baseline MAP (mm Hg)	79 (3)	76 (4)
Baseline RPP (mm Hg·min^−1^)	16090 (373)	15100 (820)

Continuous data presented as mean (SEM).

Gender presented as proportion.
